# Development of Universal Influenza Vaccines Targeting Conserved Viral Proteins

**DOI:** 10.3390/vaccines7040169

**Published:** 2019-11-01

**Authors:** Seyed Davoud Jazayeri, Chit Laa Poh

**Affiliations:** Centre for Virus and Vaccine Research, School of Science and Technology, Sunway University, Subang Jaya 47500, Malaysia; sjazayeri@sunway.edu.my

**Keywords:** seasonal influenza vaccine, universal influenza vaccine, humoral and cell-mediated immunity, conserved viral proteins

## Abstract

Vaccination is still the most efficient way to prevent an infection with influenza viruses. Nevertheless, existing commercial vaccines face serious limitations such as availability during epidemic outbreaks and their efficacy. Existing seasonal influenza vaccines mostly induce antibody responses to the surface proteins of influenza viruses, which frequently change due to antigenic shift and or drift, thus allowing influenza viruses to avoid neutralizing antibodies. Hence, influenza vaccines need a yearly formulation to protect against new seasonal viruses. A broadly protective or universal influenza vaccine must induce effective humoral as well as cellular immunity against conserved influenza antigens, offer good protection against influenza pandemics, be safe, and have a fast production platform. Nanotechnology has great potential to improve vaccine delivery, immunogenicity, and host immune responses. As new strains of human epidemic influenza virus strains could originate from poultry and swine viruses, development of a new universal influenza vaccine will require the immune responses to be directed against viruses from different hosts. This review discusses how the new vaccine platforms and nanoparticles can be beneficial in the development of a broadly protective, universal influenza vaccine.

## 1. Introduction

Influenza viruses possess single-stranded negative-sense viral RNA (vRNA) genomes belonging to the Orthomyxoviridae family. Influenza A and B genomes each comprise eight segments, whilst influenza C and D have seven segments that encode for at least 10 viral proteins [1]. Influenza A, B, and C are able to infect humans, with influenza A and B being the most common circulating types. The virus possesses a host cell-derived envelope membrane carrying the hemagglutinin (HA), the neuraminidase (NA) glycoproteins, and the ion channel matrix protein 2 (M2). Inside the envelope is the matrix protein layer consisting of the viral matrix protein 1 (M1) and the core viral ribonucleoprotein (vRNP) complexes comprising the RNAs and the binding proteins, including the heterotrimeric polymerase complex (PA, PB1, PB2) and multiple copies of nucleoproteins (NP) [2].

Influenza virus can cause seasonal or pandemic influenza infections [3]. In the United States, the Centers for Disease Control and Prevention (CDC) estimates that influenza has caused 9.3 to 49 million infections, 140,000 to 960,000 hospitalizations, and 12,000 to 79,000 deaths annually since 2010 [4]. Iuliano et al. (2018) estimated the global seasonal influenza-associated respiratory deaths annually to be 291,243 to 645,832 [5]. These global influenza-associated mortality estimates are higher than those previously reported by World Health Organization (WHO) (250,000 to 500,000) [6], which suggested the previous estimates might have underestimated the disease burden. A total of 186 pediatric deaths in the United States were recorded in the CDC report during the 2017–2018 influenza season and this number exceeded the previous 171 deaths reported during the 2012–2013 season. Approximately 80% of deaths in the United States happened in children who did not receive seasonal influenza vaccinations [7].

Influenza pandemics caused by influenza A viruses can also emerge at unpredictable intervals which could significantly increase morbidity and mortality when compared with seasonal influenza. In 1918, 1957, 1968, and 2009, four influenza pandemics have occurred and claimed millions of lives [8]. Additionally, in recent years, influenza viruses from avian (H5N1, H7N9 and H9N2), swine (H1N1, H1N2 and H3N2), and other zoonotic influenza viruses have caused more human infections and mortalities compared to the past decades [9]. Nevertheless, there is a worry that viral shifts or drifts might allow efficient transmission in humans and cause the next influenza pandemic [10].

Influenza vaccines have been in development since the early 1940s and still play a critical role in providing protection. Current commercial influenza vaccines are mainly produced by egg-based technologies and have several limitations, such as the supply of vaccine-quality eggs, vaccine manufacturers cannot be flexible in the amount of doses being produced, moderate efficacy in certain populations, time-consuming in the production process which could lead to vaccine shortages and lack of cross-reactivity, especially during pandemic situations [11]. When the representative strains selected for vaccine use are not well matched to the seasonal circulating influenza strains, the effectiveness of the seasonal vaccines becomes very low [12].

Passaging the influenza viruses in eggs might also allow for the additional mutation(s) to occur during manufacturing and could affect the efficiency of the seasonal vaccine [13]. Additionally, some people are allergic to eggs and need to be vaccinated with egg-free influenza vaccines. There are various types of influenza vaccines that are commercially available in the market. Most of the clinically-available influenza vaccines could efficiently induce antibody responses against the globular head of HA protein [14]. The effectiveness of seasonal influenza vaccine was reported to range between 10% and 60% [15]. In 2017, the overall efficacy of the seasonal influenza vaccine for Australia was reported to be 33% [16] and for the United States was 36% [17].

Influenza viruses are subjected to continuing mutations each season and the current seasonal influenza vaccination strategy keeps us at least one year behind the evolving virus [10]. The newly emerged pandemic influenza virus could lead to making a matched monovalent vaccine that might never be utilized, such as the H7N9 and H5N1 stockpiles which have since drifted or deteriorated [10]. Currently, there is a crucial need to develop more effective “broadly-reactive” or “universal” influenza vaccine with high protection efficacy against new potential seasonal and pandemic strains.

Several studies have shown that humoral responses to HA and NA antigens could play an essential role in host immunity against influenza due to antibodies, especially anti-HA could neutralize the virus [18,19,20]. However, pre-existing CD8 T cell responses against the relatively conserved internal antigens of the influenza virus are highly desirable as they could elicit immune responses towards multiple viral strains, providing higher cross-protection capability in the absence of neutralizing antibodies [21].

Advances in influenza biology, immunology, vaccinology, and bioinformatics have allowed the contribution of new strategies to the rational design of universal influenza vaccines. Due to the conserved antigen epitopes among distant virus strains, the development of a universal influenza vaccine is theoretically possible. Here we review the commercial influenza vaccines, different arms of the immune responses, and variable as well as conserved viral targets, and discuss recent scientific progress in the development of virus-like particles (VLPs), nucleic acid (DNA and mRNA), and peptide-based vaccines as potential next-generation universal influenza vaccines.

## 2. Commercial Influenza Vaccines

Strains used in licensed seasonal influenza vaccines are selected in February and September following the influenza seasons in both the northern and southern hemispheres, respectively. The WHO makes recommendations for next seasonal influenza vaccines based on the compositions of viruses from the surveillance, laboratory, and clinical observations [22]. Three classes of licensed influenza vaccines are commercially available: Detergent-split inactivated influenza vaccine (IIV), recombinant influenza vaccine (RIV), and live attenuated influenza vaccine (LAIV) (Table 1).

Inactivated vaccines (most frequently used for the prevention of influenza) include whole inactivated virus vaccines (WIV), split virus vaccines, subunit vaccines, and virosomal influenza vaccines. Typically, WIV production starts with influenza virus growth in 11-day-old pathogen-free embryonic chicken eggs, followed by chemical inactivation with β-propiolactone or formaldehyde. The allantoic fluid is harvested and clarified by centrifugation and purification. Instead of inactivation by chemical agents, Furuya et al. (2010) advocated γ-irradiation to inactive influenza A. They demonstrated cross-protective immunity being induced by intranasal vaccination of mice with γ-irradiated influenza A virus was mediated mainly by CD8 T cell responses [23,24].

Regarding the split virus vaccines, the virus is split before formulation and filtration, leading to the removal of the detergent. Influenza subunit virus vaccines require further purification steps before the final formulation to remove the nucleocapsids and lipids [22,25].

RIV and FluCelvax (IIV) are 100% egg-free influenza vaccines which were authorized for use in the United States in 2013 and 2016, respectively, for people with egg allergies. The WHO has selected the HA protein antigens from three influenza virus strains annually and each of the three recombinants HAs were expressed through a viral host (baculovirus *Autographa californica* nuclear polyhedrosis virus) in the insect cell line. The three individual HA proteins extracted from the cells in the presence of detergent were further purified by column chromatography [26,27]. RIV has a shelf life slightly shorter than most other injectable influenza vaccines currently available and expires within nine months from the date of production [28].

LAIV for human application was developed in the 1960s independently by the United States and Russia after serial passages of the influenza virus in eggs [22]. Reverse genetics was used to generate multiple mutations in influenza viral genes to produce temperature-sensitive and cold-adapted LAIV. The cold-adapted vaccine viruses could only replicate and grow when the temperature was below 25 °C and stopped growing when the temperature was above 37.8 °C [29]. FluMist, a cold-adapted LAIV, was initially licensed in the United States in 2003 as an intranasal trivalent LAIV for use in people aged two to 49. In 2012, it was replaced with the quadrivalent LAIV (LAIV4). The Advisory Committee on Immunization Practices (ACIP) recommended that LAIV4 should not be used during the 2016–2017 and 2017–2018 influenza seasons due to poor efficacy against influenza A(H1N1)pdm09-like viruses circulating in the United States during the 2013–2014 and 2015–2016 seasons [30]. Investigations into the possible reason for this low efficacy against influenza A(H1N1)pdm09 demonstrated the replication of viruses in human nasal epithelial cells was reduced when compared with pre-pandemic influenza A(H1N1) LAIV viruses [31].

A new A(H1N1)pdm09 influenza virus such as the A/Slovenia/2903/2015 strain was included in the LAIV4 formulation to replace the A/Bolivia/559/2013 strain for the 2017–2018 season. The ACIP again suggested LAIV4 as a vaccine alternative for the 2018–2019 season on 21 February 2018 [30].

Regardless of the influenza vaccine strains or manufacturing platforms, most of the current commercial influenza vaccines are generated by the growth of the selected viruses in embryonic chicken eggs that depend on the continuous supply of chicken eggs and, in recent years, influenza viruses have not been reported to grow well in eggs [22]. Forcing the selected viruses to grow in embryonic chicken eggs frequently results in egg-adapted modifications associated with some antigenic mismatches that could lead to changes in the HA head region and reduce the efficacy of vaccine-generated antibody responses. This has been an issue for the H3N2 component of the vaccine for several recent influenza seasons [22,32].

The general vaccine efficacy in the 2014–2015 influenza season was only 19% in the United States, while in the 2017–2018 influenza season, during the most severe outbreak of an influenza epidemic, the vaccine effectiveness was only 25% [15,33]. This was due to the emergence of the H3N2 virus which was different from the vaccine strains present in the influenza vaccine formulation. 

## 3. Immune Responses Required for Universal Influenza Protection

**Figure 1:**
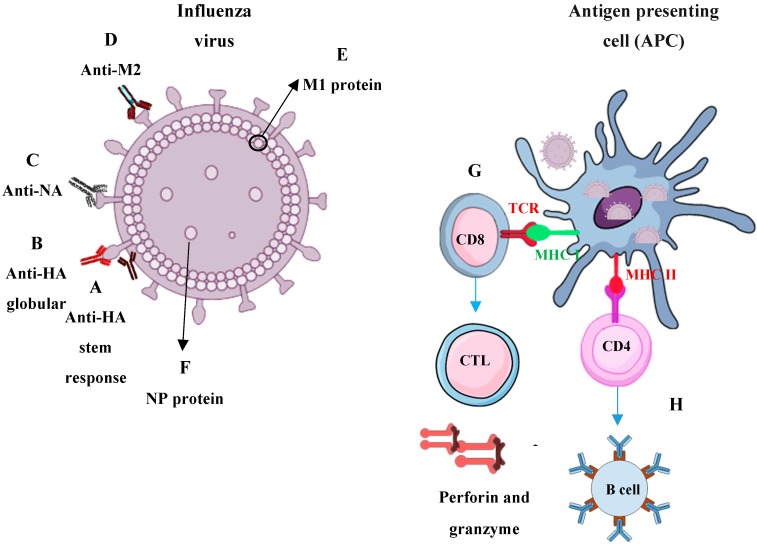
Antigens for universal influenza vaccine development. (**A**) Neutralizing antibodies against highly conserved hemagglutinin (HA) (major protein of influenza virus) stem can provide broadly protective immune responses and cross-protection. (**B**) Neutralizing antibodies against the globular head of HA can prevent virus binding to sialic acid and prevent the conformational change that leads to fusion. (**C**) Anti-neuraminidase (NA) (second major protein of influenza virus) response targets the enzymatic site to prevent virus entry, inhibit replication efficiency, decrease disease severity after infection and cross-protection. (**D**) Anti-matrix protein 2 (M2) antibodies (third major protein of influenza virus) provide a better cross-protective response due to the high conservation. Non-neutralizing Ab against this domain mediates its mediates protection by antibody-dependent cell-mediated cytotoxicity. (**E**) matrix protein 1 (M1) is an internal protein which is generally not exposed outside of the virus and needs to be processed by major histocompatibility complex I (MHC I) for CD8 T cell antigen recognition. (**F**) Highly conserved nucleoproteins (NP) viral proteins being used as a target to CD8 T cells to provide better protection from several infections. (**G**) CD8 T cells recognize peptides derived from variable (HA and NA) and highly conserved internal proteins (NP and M1) presented by MHC I at the surface of antigen presenting cell (APC)/infected cells via their T cell receptor (TCR). Cytotoxic T lymphocytes (CTL) release cytotoxic granules containing perforin and granzymes which lysis the infected cells. (**H**) Memory CD4 T cells are required for early Ab and CD8 T cell recruitment responses.

Host immune responses against influenza virus are multifactorial and rapid mutations allow the viruses to escape the immune responses generated after seasonal vaccination or infection. Therefore, a promising universal influenza vaccine needs to stimulate B, CD8, and CD4 T cell responses against various conserved proteins for efficient viral clearance, long-lasting immunity, and prevention of reinfection (Figure 1).

The influenza HA and NA surface proteins are the major targets of humoral immune responses and most of the current commercial seasonal influenza vaccines are targeting the globular head of HA for neutralization. Rapid antigenic variations due to the accumulation of mutations within the antibody-binding sites of HA and NA of circulating viruses have abrogated the affinity of some antibodies to the viruses. Alternatively, the stem of HA is highly conserved when compared with the globular head which made it a strong target for vaccine development to induce broadly neutralizing antibodies and protective immune responses. Thus, targeting any conserved sites in the HA stem could be a promising approach to the development of a new universal vaccine [40]. However, the immunodominance of the head and steric hindrance of the stem could make it more difficult to mount immune responses [38]. Thus, inducing broadly-neutralizing anti-stem antibodies remains a challenge [41]. In addition to HA and NA humoral immune responses, some other conserved influenza viral antigens could be recognized by the CD8 T cells and they are mostly found in the inner M1, M2, NP, PA, and PB1 proteins [42]. Vaccines based on these antigens were heterosubtypic and were able to induce broad immune responses as well as protection against viruses from different hosts in animal models [43,44,45]. When there are no protective antibody responses to newly evolved influenza viruses, T cells could provide heterosubtypic immunity to various influenza virus subtypes and even unrelated viruses because of conserved peptide homology [46,47]. Therefore, the activated T cells could provide the required immunological protection from infection with influenza. In the absence of effective neutralizing antibodies, CD8 T cells were able to prevent the viral spread and conferred high cross-protection against heterologous influenza viruses strains in both mice and humans [48,49,50]. Although CD4 T cell responses to infection with influenza and vaccine development have not received as much attention as the CD8 T cells and humoral responses, their role is still important for host protection against influenza [51]. In fact, depletion of CD4 T cells at the time of vaccination led to the loss of all the protections; therefore, CD4 and CD8 T cells played important protective role during the challenge. In addition, CD4 T cells demonstrated a necessary role during influenza vaccinations and provided cross-protective anti-influenza immune responses.

Pre-existing antibodies failed to neutralize various influenza viruses but CD4 memory T cells were able to help in directing a faster antibody response via cytokine secretion in response to mutated or immunologically novel viral antigens. There was limited virus shedding and disease severity, possibly due to the direct lysis of infected epithelial cells [51,52,53]. Sheikh et al. (2016) demonstrated cross-protection could be achieved by activating CD4 and CD8 T cells against highly conserved regions of the influenza proteins [54].

A vaccine that could induce all of these immune responses against the influenza virus probably would overcome most of the limitations of the current seasonal influenza vaccines and become a universal influenza vaccine in the future.

## 4. Potential Universal Vaccine Platforms

**Figure 2:**
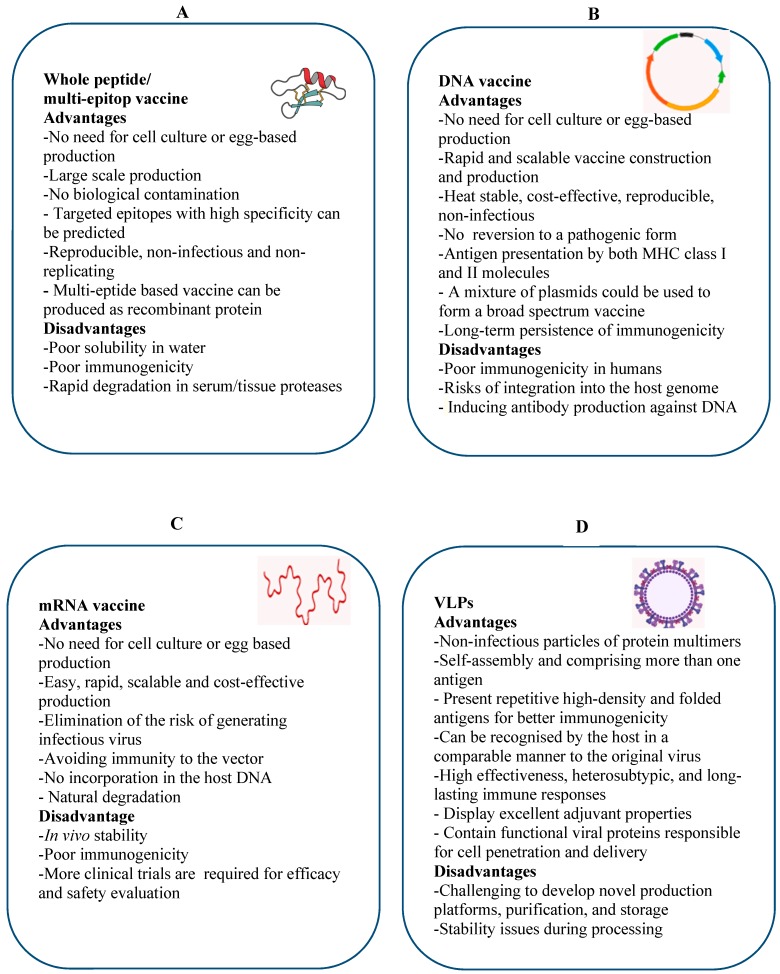
Advantages and disadvantages of whole peptide/multi-epitope (**A**), DNA (**B**), mRNA (**C**), and virus-like particles (VLP) (**D**) vaccine platforms for developing a novel universal influenza vaccine: All four vaccine platforms are able to induce humoral and cellular immune responses.

An ideal universal influenza vaccine will be effective against all influenza A and B viruses regardless of any antigenic mutation(s) or HA and NA subtypes. The objective of this vaccine is to induce cross-protective and long-lasting immune responses by stimulation of both arms of the immune system. To design this type of vaccine, the highly conserved epitopes present in HA, NA, or M2 and internal proteins such as NP and M1 should be targeted to induce cross-protective antibodies and T cells. Development of a new universal influenza vaccine can be significantly facilitated by newly-developed platform technologies such as multi-epitope, VLP, DNA, and mRNA-based vaccines, which will be discussed. Advantages and disadvantages of whole peptide/multi-epitope (Figure 2A), DNA (Figure 2B), mRNA (Figure 2C), and VLP (Figure 2D) vaccine platforms including their production strategy, immunogenicity, and stability are discussed in Figure 2.

### 4.1. Multi Epitope-Based Vaccine

The epitope-based approach focuses on conserved sequences of an antigen with the minimal components that activate the lymphocytes: short peptides of eight to 10 amino acids for activation of the CD8 T cells [55], 12 to 16 amino acids for activation of the CD4 T cells [56], and longer peptides of up to 85 amino acids for activation of the B cells [57]. 

Recently, Rodriguez et al. (2018) developed a multi-peptide-based vaccine (Vacc-FLU) combining one M2e peptide eliciting the humoral and three peptides representing the M2, NP, and a mixed M2 and NP that were capable of eliciting the cell-mediated immune responses by using ISA51 as an adjuvant in the murine model. Following two subcutaneous vaccinations, humoral and cellular immune responses were induced to levels that could prevent symptomatic influenza infections and provided partial protection in mice [58].

The stem region of the HA protein is considered as highly conserved when compared with the globular head domain and could be considered as a promising target for a novel universal influenza vaccine. Lu et al. (2017) identified a new mouse CD4 T cell epitope in the HA stem domain of the pandemic H1N1 influenza virus. The intraperitoneal vaccination of mice with aluminum hydroxide gel as an adjuvant induced significant polyfunctionality of splenocytes and degranulation. Furthermore, immunized mice with the CD4 T cell epitope exhibited interindividual sharing of the CD4 TCRβ clonotypes, and demonstrated partial protection against a lethal pandemic H1N1 challenge [59]. Two influenza vaccines, the pentavalent Wyeth/IL-15/5Flu and the bivalent modified vaccinia virus Ankara (MVA)/IL-15/HA/NA, were able to induce cross-neutralizing antibodies and conferred cross-clade protection when challenged with the highly pathogenic avian influenza H5N1 virus of a different clade [53].

#### 4.1.1. Recombinant Epitope-Based Vaccine

The epitope-based Multimeric-001 (M-001) was firstly explored by Ben-Yedidia et al. [60] and then developed by BiondVax Pharmaceuticals Ltd. (Ness Ziona, Israel). M-001 is a single recombinant protein that consists of three repetitions of nine conserved linear epitopes from HA (four B and one CD4 T cell epitopes), NP (two CD8 T cell and one CD4 T cell epitopes), and M1 (one peptide containing both B and CD8 T cell epitopes), which were derived from influenza A and B strains expressed in *Escherichia coli* (*E. coli*). Preclinical and clinical studies of the M-001 confirmed the protection of mice against different influenza strains by inducing both B and T cell-specific immune responses. As M-001 did not contain any variable epitope of the HA of influenza, it was not able to induce specific hemagglutination inhibition (HAI) antibodies and needed to be boosted with a seasonal or pandemic strain [61,62]. The phase II clinical trial employed two priming doses of M-001, followed by a seasonal quadrivalent inactivated influenza vaccine (IIV4). The 24-month clinical trial from April 2018 was conducted to evaluate the safety, reactogenicity, and immunogenicity of the combined vaccines, which is still under progress in three United States sites [63].

Another recombinant protein based on M2 ectodomain (M2e) was produced in *E. coli* by the fusion of two tandem copies of consensus M2e sequences from human influenza A and two copies of M2e from avian A/H5N1 viruses linked to the flagellin of *Salmonella typhimurium*. Intranasal immunization of mice with the recombinant protein resulted in significant humoral and cellular responses and provided comparable protection against lethal challenges with human H1N1/H3N2 and avian H5N1 influenza A viruses [44]. Another recombinant protein, Flg-HA2-2-4M2e, was designed with a combination of *Salmonella typhimurium* flagellin as a carrier with the conserved M2e and influenza A HA stalk epitopes. Intranasal immunization of mice followed by two further boosts demonstrated that the Flg-HA2-2-4M2e recombinant protein was highly immunogenic, and stimulated both systemic and mucosal immune responses with the production of lung-resident virus-specific effector memory T cells. The Flg-HA2-2-4M2e vaccine candidate was cross-protective and induced antiviral antibody production against various phylogenetic groups of viruses [64]. Early-stage clinical studies have demonstrated that the M2e-based vaccine was safe and was able to generate an anti-M2e antibody in up to 90% of healthy volunteers [65,66]. However, further clinical development of M2e as a universal influenza vaccine did not progress well due to the rapid decline of anti-M2e IgG titers over time [67].

#### 4.1.2. Recombinant Vectored Epitope-Based Vaccine 

The conserved antigens of H1N1 and H5N1 influenza viruses from 1970 until 2010 were analyzed for all hosts and countries by Huber et al. (2015) [68]. The influenza antigens NP, PB1, and M1 have been selected as they have the largest amount of conserved regions, which also included a number of recognized CD8 and CD4 T cell epitopes. Vaccination of mice and ferrets with the conserved B and T cell epitope-based vaccine showed specific antibodies that could reduce viral loads in the lungs [68]. Some peptides present in the proteins of the influenza virus are conserved in humans and animals, which could be expected to remain conserved even after genetic re-assortment. To develop a novel universal influenza vaccine, it is vital to identify sequences of conserved antigens that include both human and animal (bird and swine) B and T cell epitopes. Recently, Tutykhina et al. (2018) analyzed the sequences of M2 and NP collected from influenza A over the last 100 years, with respect to mutations in the sequences of recognized B and T cell epitopes, to determine conserved and evolutionarily significant epitopes. Finally, consensus sequences of M2 (MSLLTEVETPIRNEWGCRCNDSSD and SLLTEVETPIRNEWGCRCNDS) and NP (ILRGSVAHK and KTGGPIYRR) were found to be 100% coincident with influenza viruses H1N1, H3N2, and H5N1 [69]. A recombinant adenoviral vectored vaccine included nucleotide sequences corresponding to epitope-enriched amino acid sequences of M2 and NP, representing B and T cell epitopes of human, swine, and avian origin.

A single intranasal vaccination of mice with the recombinant Ad5-tet-M2NP vaccine induced robust specific cellular responses to each of the encoded antigens and demonstrated an extremely wide-ranging protection efficacy following challenge with five different influenza A viruses originating from three different hosts. Eight months after immunization, high levels of effector memory CD44^+^CD62^−^ T cells against M2 and NP were found in splenocytes. This finding indicated that conserved B and T cell epitopes might become one of the main approaches for developing a universal influenza vaccine.

Continual variations in the influenza virus antigen genes are the main cause of influenza outbreaks and pose challenges for the development of a universal influenza vaccine. Consequently, epitope predictions and utilization are of value in the design of highly conserved multi-epitope as recombinant universal influenza vaccine [70,71].

### 4.2. DNA-Based Vaccine

Since the 1990s, efforts to develop influenza DNA vaccines have been on-going. Despite original enthusiasm about protection in small animal models, studies in larger animal models have met with relatively poor efficacy. Recent advances in DNA vaccine development showed that the poor efficacy could be partially overcome by improving the selection of antigens and expression vectors, vaccine delivery methods, routes of vaccination, targeting APC, co-delivery of adjuvants, and prime-boost strategy [72,73].

To increase the effectiveness of the DNA vaccine and improve the immune responses, HA from H7N1 was targeted to either major histocompatibility complex II (MHC II) molecules or chemokine receptors 1, 3, and 5 that were displayed on APC. APC-targeted HA as a DNA vaccine was able to considerably increase the level of neutralizing antibodies in sera and specific CD8 T cells, and protected mice against a lethal H7N1 challenge [72].

In another study, Alexander et al. (2010) used fifty-two diverse influenza A sequences from strains currently in circulation (H1N1, H3N2), strains from zoonotic infections of humans (H1N1, H5N1, H7N2, H7N3, H7N7, H9N2) and past pandemic strains (H1N1, H2N2, H3N2) for CD4 T cell epitope conservancy analysis. Vaccination with the conserved CD4 T cell influenza-specific epitopes was able to elicit peptide-specific immune responses and T cell memory responses, which led to more rapid and robust induction of HA-specific antibody responses. Peptide-specific immune responses protected mice from lethal mouse-adapted H1N1 PR8 influenza virus challenge [74]. A polyvalent H5 DNA vaccine from H5N1 influenza viruses of A/Vietnam/1203/04, A/Anhui/1/2005, A/Indonesia/5/2005, and A/Hongkong/156/97 was evaluated in New Zealand white (NZW) rabbits and the elicited antibodies could provide broad protective humoral responses against various key H5N1 viruses [75].

Cynomolgus macaques were vaccinated with an influenza multi-antigen DNA vaccine containing the HA, NP, and M2 proteins. Following a heterologous pandemic H1N1 strain challenge, immunized animals showed faster virus clearance, significantly lower viral loads, elicited robust serum/mucosal antibodies, and strong cross-reactive cellular immune responses, which could mediate significant cross-protection [76].

Karlsson et al. (2018) vaccinated pigs twice with a polyvalent influenza DNA vaccine encoding HA, M, NA, and NP on the dorsal side of the back by using a needle-free intradermal application of liquid devices. Two weeks after the second immunization, all the animals were intranasally challenged with the 2009 pandemic H1N1 and none of the vaccinated pigs became infected while all the control pigs were infected. The results showed that a DNA vaccine could elicit HA and NA inhibiting antibodies and cross-reactive neutralizing antibodies in a dose-dependent manner [77].

Recently, the first phase 1 clinical trial of a trivalent HA DNA vaccine against seasonal influenza was conducted in the United States. Children from six to 17 years old received DNA vaccines intramuscularly by the needle-free jet injector (Medical Technologies, Inc., Tualatin, OR, USA) and boosted with the split virus 2012/2013 seasonal trivalent inactivated influenza vaccine (IIV3) approximately 18 weeks later. The HA DNA vaccine prime-IIV3 boost was safe and well-tolerated compared to the prime-IIV3-boost-IIV3 regimen. Overall, humoral responses were comparable among groups although the increase of HAI antibodies to A/California/07/2009 [A(H1N1)pdm09] was considerably higher after vaccination with the HA DNA-IIV3 when compared to the IIV3-IIV3 [78]. After more than two decades of research, the first commercial DNA vaccine against the highly pathogenic avian H5N1 in chickens has been provisionally approved in 2017 by the United States Department of Agriculture (USDA) [79]. Successful control of influenza can only be achieved through more in depth understanding of the inter-relationships between animal and human influenza vaccines. This could lead to the design of a universal influenza vaccine. Although a DNA-based vaccine against influenza is a flexible platform with long-term persistence and cross-protective efficacy in preclinical challenge models, it has yet to show potency in human clinical trials. 

### 4.3. mRNA-Based Vaccines

mRNA vaccines are comprised of antigen-encoding genes whereby, after administration into host cells, the RNA is translated into proteins that could elicit protective immunity against the infection. Unlike DNA vaccines that need to enter into the cell nucleus, mRNA-based vaccines are translated in the cytoplasm directly [80]. Although mRNA-based vaccines are very promising, there are limitations associated with delivery and stability issues. Currently, two types of mRNA-based vaccines are being created: the self-amplifying mRNA and the conventional non-amplifying mRNA [81].

The self-amplifying mRNA is capable of self-replication by RNA-dependent RNA polymerase, producing various copies of the antigen-encoding mRNA, and expressing high levels of the heterologous genes in the host cytoplasm. Conventional mRNA vaccines contain an open reading frame for the target antigen, which is flanked by untranslated regions, and have a terminal poly(A) tail, which drives transient antigen expression after transfection [82].

Magini et al. (2016) demonstrated immunization of mice with self-amplifying mRNA expressing conserved internal influenza NP and M1 antigens, delivered individually or in conjugation with lipid nanoparticles (LNanoPs), was able to induce strong polyfunctional CD4 T cells as well as central and effector memory CD4 and CD8 T cells. The co-administration of self-amplifying mRNA-encoding NP and M1 antigens along with the monovalent inactivated influenza vaccine (MIIV) could induce, simultaneously, HA, NP, and M1-specific T cells, HA-specific neutralizing antibodies, and also improved MIIV efficiency against a heterologous challenge [83]. 

The timing of the production and availability of influenza vaccines is essential for an effective response to new seasonal influenza outbreaks. It could take at least six months to formulate a new FDA-approved pandemic influenza vaccine that could leave the population without any protection [84]. In 2009, after the disease peaked, the seasonal influenza vaccine became available and this has motivated the development of a rapid vaccine that is important to protect against newly evolved strains. Hekelen et al. (2013) developed the self-amplifying mRNA expressing the H7 vaccine against H7N9 only eight days after the gene sequence became available. Therefore, the mRNA-based vaccine has the ability to provide the technique for the fast development of an efficient next-generation influenza vaccine in humans [85].

Lutz et al. (2017) showed a single intramuscular vaccination with the A/Netherlands/602/2009 (H1N1) HA-encoding mRNA formulated in LNanoPs, which induced >1:40 HAI titers in mice. Administration of a second dose (three weeks after the prime vaccination) strongly boosted immune responses and resulted in >1:160 HAI titers for up to one year that remained stable in all animals. Fluad^®^ is a commercial inactivated influenza vaccine containing the HA and NA of influenza virus strains H1N1, H3N2, and B/Brisbane with MF59C.1 as the adjuvant. A single dose of the H3N2-HA mRNA/LNanoP vaccine was effective to induce above 1:40 H3N2-HI titers and was comparable to antibody titers induced by Fluad^®^ [86].

Pardi et al. (2018) evaluated the effectiveness of a newly-developed influenza vaccine using nucleoside-modified and purified mRNA-encoding full-length HA protein formulated in LNanoPs. The results demonstrated that vaccination with HA mRNA-LNanoPs could induce strong specific humoral immune responses against the conserved HA stalk domain in mice, ferrets, and rabbits. The HA stalk specific antibodies protected mice against A/California/07/2009 H1N1,(homologous), A/Puerto Rico/8/1934 H1N1 (heterologous), and H5N1 (heterosubtypic) influenza virus infection [87].

The self-amplifying mRNA expressing HA influenza virus when formulated with oil-in-water cationic nanoemulsion showed immunogenic response in ferrets and facilitated containment of viral replication in the upper respiratory tract. It was able to induce functional neutralizing antibodies and HA-specific T cells in mice [88]. Furthermore, vaccination with an HA-encoded mRNA vaccine conferred long-term immunity and elicited B and T cell-dependent protection in the newborn and 18-month-old mice, indicating that the mRNA vaccine was capable of reducing the burden of influenza disease at the extremes of age [89].

mRNA-based vaccines against influenza provide a rapid, scalable, and cost-effective production platform with no incorporation in the host DNA. It is also able to induce humoral and cellular immune responses (Figure 2). The effective delivery of mRNA vaccines into the cytoplasm of host cells within acceptable limits of tolerability is a key aspect to improve the vaccine efficacy [90].

### 4.4. Virus-Like Particles (VLPs)

VLPs are non-infectious particles obtained from the self-assembly of viral structural proteins carrying viral antigens that could mimic the structure of live virus particles. Although most of the commercial influenza vaccines are still based on the inactivated embryonic egg-grown virus, advances in cell culture technologies involving plant, insect, and mammalian cells could provide egg-independent manufacturing options to produce VLP vaccines [34]. In preclinical and clinical studies, VLPs have been effective in their safety and efficacy. The VLPs were highly immunogenic and could activate both cellular and humoral immune responses. Currently, two VLP-based vaccines against hepatitis B virus (HBV) and human papillomavirus (HPV) are licensed for human use [91].

The influenza VLP vaccine derived from the A/Udorn 72 (H3N2) strain expressing HA and M1 generated in baculovirus was able to confer 100% protection in mice against the heterologous A/Hong Kong/68 (H3N2) virus [92]. A vaccine comprising three tandem copies of M2e, NP, and the HBV core (HBc) in VLPs could induce robust antibody and T cell immunity, improving the protection against a lethal challenge with the pandemic 2009 H1N1 and the highly pathogenic avian influenza (HPAI) H5N1 virus in mice [93]. Most VLP-based influenza vaccines were developed comprising a single antigen or a combination of HA, NA, and M antigens. 

VLPs comprising a mixture of H1, H3, H5, or H7 HAs were produced using a recombinant baculovirus expression system administered intranasally and then showed effective protection in mice when challenged with influenza viruses expressing the 1918 H1, 1957 H2, avian H5, H6, H7, H10, and H11 HA subtypes. Broad protection against various influenza A subtypes suggested that the VLP platforms are promising and practical approaches for universal influenza vaccine development [94]. 

Several clinical trials in distinct populations (children, adults, and elderly) showed that significant antibody titers induced by commercial egg-based seasonal influenza vaccine were detectable up to 18 months after the prime immunization. The performance of an insect cell-derived pandemic H1N1 2009 influenza VLP vaccine was successfully demonstrated in human clinical studies and showed the persistence of antibodies up to 24 months after vaccination [95]. Subjects who were re-vaccinated with a subsequent inactivated influenza virus vaccine induced the highest antibody titers to the pandemic strains when compared to the unvaccinated subjects.

Luo et al. (2018) vaccinated mice with heterosubtypic influenza HA VLPs containing H1, H8, and H13, or H3, H4, and H10 in various combinations using baculovirus expression systems. Multiple antigenic variants with high genetic diversity could mimic the natural structure of the stalk domain and generated broader humoral responses to confer complete protection against different homosubtypic and heterosubtypic influenza viruses [96]. Although studies on influenza surface viral proteins have shown promising results for developing universal vaccines, M2e as a naturally-conserved protein has been included in VLPs and showed enhanced cross-protection against different influenza virus subtypes like the H1N1, H3N2, and H5N1 [97,98]. 

In March 2013, Avian H7N9 influenza A virus was first recorded in China and the ability to transmit from poultry to humans raised serious concerns about its pandemic potential. Intramuscular vaccination of chickens and mice with VLPs comprising multi-antigens such as the HA, NA, and M1 of avian influenza A (H7N9) expressed in baculovirus elicited HI serum titer and antibodies against NA and M1 proteins [99]. In chickens, after two subcutaneous vaccinations with H5, H7, and H9 VLPs using the baculovirus expression system, cross-reactive immune responses, and protection were observed when challenged with heterologous HPAI H5N2, H7N3 and low pathogenic avian influenza H9N2 [100]. In pigs, VLPs encoding HA, NA, and M1 proteins of H1N1pdm09 produced by baculoviruses were found to elicit strong IgG (serum), IgA (mucosal), and viral neutralizing antibodies. Following challenge with the pandemic H1N1 2009 strains, the vaccinated pigs were protected and lung lesions were decreased; virus shedding and virus replication in the lungs were also inhibited [101].

In a human challenge study with the H3N2 virus, treatment with a monoclonal M2e specific antibody was found to lead to a fast recovery. However, in phase I clinical study with the M2e VLP vaccine, there was a rapid decline in anti-M2e IgG. New development of the M2e VLP vaccines could involve the combination of a prime boost strategy with an HA-stalk based vaccine. Alternatively, headless HA stalk domains could be incorporated and co-expressed [65]. The new generation of VLP vaccines could involve incorporation of multiple conserved epitopes such as HA, NP, and M to confer more cross-protection.

Plant-based VLP vaccines against influenza have proven to be immunogenic in both animals and humans and have the potential to address several limitations of current vaccines including response time and scalability in the event of a pandemic [102,103]. The Medicago VLP vaccine is a quadrivalent influenza vaccine comprising recombinant H1, H3, and two B hemagglutinin proteins derived from 2018–2019 influenza virus strains. The phase III clinical trial of the Medicago VLP vaccine has been in progress to assess the efficacy, safety, and immunogenicity in adults 65 years and older [104]. In the study reported by Hodgins et al. (2019) a novel plant-derived VLP bearing HA could induce a balanced humoral and cellular response in old mice [105]. Since the plant-based VLPs carried only HA alone, it might not provide the protection to all clades of influenza. The recombinant baculovirus expression system in insect cells appeared to have high flexibility to produce universal VLP vaccines containing multiple antigens (HA, NA, M1, and M2) and could provide broad protection. 

## 5. Nanotechnology and Influenza Vaccine Development

Some promising results have been shown by epitope-based vaccines to induce protective immune responses. The highly conserved epitopes across different influenza virus strains have been extensively used to formulate universal influenza vaccines from either humans and animals. Conserved antigen-based vaccines suffer from low immunogenicity and in most cases resulted in a weak immune response with partial protection. These epitope-based vaccines require boosting and adjuvants for robust and long-lasting immune responses. Due to the fast degradation of subunit vaccines in the body, encapsulation and effective delivery are challenges for the universal influenza vaccine development. Based on the recent developments in chemical engineering and biological understanding, the new generation of NanoP-based vaccines can potentially overcome the low immunogenicity, delivery, and controlled release and lead to enhancement of antigen presentation to achieve desired immune responses.

NanoPs are compatible with a variety of immunogens and universal influenza vaccine development has intensively used new approaches of nanotechnology. The morphological and structural properties of NanoPs significantly improved the immunogenicity of conserved influenza viral antigens which induced long-lasting immunity [106]. NanoPs could mimic the biophysical and biochemical characteristics of virions and initiate strong signals to enhance innate immunity as well as eliciting robust cellular and humoral immune responses with minimal cytotoxicity [107].

Aluminum-based mineral salts are the oldest and most widely used adjuvant in influenza vaccine development [108]. Aluminum-based adjuvant may be sufficient to elicit antibody responses with acceptable safety and efficiency, but they are weak immunostimulators of cellular immune responses and are limited in their applications against intracellular pathogens vaccines [109]. NanoPs are increasingly used as an adjuvant (nano-adjuvant) for vaccine development and formulations due to their biocompatibility and flexibility in shape, size, and physicochemical properties. Nano-adjuvants could improve the antigen delivery and presentation by professional APC and act as immunostimulators that could activate innate and adaptive immunity. However, NanoPs have their own technical issues and limitations such as loading of sufficient dose of the immunogen in the NanoPs, encapsulation of the immunogen, and antigen denaturation during encapsulation [110].

Deng et al. (2018) generated a double-layered protein NanoP by desolvating tetrameric M2e peptides into the protein nanoparticle cores and coating these cores by crosslinking headless HAs. Intramuscular vaccinations of mice with protein nanoparticles were able to induce robust long-lasting immune responses and completely protected mice against divergent influenza A viruses. These findings demonstrated the significance of incorporation both headless HA stalk domain and M2e as a potential universal influenza vaccine [111].

A combination of conserved B and T cell peptides of human influenza A virus H1N1 and norovirus P particle-containing M2e (M2e-PP) from swine influenza viruses were encapsulated using polylactic-co-glycolic acid (PLGA) NanoPs and administered intranasally. The results demonstrated that PLGA NanoPs mediated the delivery of conserved peptides, induced the virus-specific cellular responses in the lungs, reduced the viral titer of the heterologous influenza virus used in the challenge, which resulted in robust prophylactic protection in pigs [112]. However, the encapsulated conserved peptides with PLGA did not improve humoral immune responses (mucosal IgA and systemic antibodies) in pigs. In another study, Dhakal et al. (2018) used liposome NanoPs as a carrier to incorporate ten peptides representing the highly conserved T and B cell epitopes and delivered with monosodium urate crystal as adjuvant. Intranasal immunization of pigs with liposomal delivery of conserved peptide vaccines improved specific cellular and mucosal humoral immune responses [113].

Moreover, Bahl et al. (2017) showed that a single dose of the modified HA mRNA-based vaccine of H7N9 encapsulated in LNanoPs could protect mice from a lethal influenza challenge as well as decreased lung viral titers in ferrets. In a human escalating-dose phase 1 H10N8 study, very high seroconversion rates were observed. The results suggested that modified mRNA formulated with LNanoPs could induce protective immunogenicity against influenza infection with acceptable tolerability profiles [114]. Therefore, liposomes could encapsulate different antigens of influenza viruses and protected humans, swine and mice from highly variant influenza serotypes. In another approach, the M2e peptide of influenza virus was conjugated to gold NanoP and co-delivered with CpG oligodeoxynucleotide as an adjuvant. Intranasal immunization of M2e-gold-CpG in mice induced activation of B cells in the lungs and strong anti-M2e IgG subtype response in serum. The lethal challenge of vaccinated mice with H1N1 (pandemic strain), H3N2, and H5N1 led to 100%, 92%, and 100% protection, respectively. Gold NanoPs as an intranasal vaccine carrier significantly increased the immunogenicity of conserved M2e peptides in mice [115]. The H1-stabilized stem ferritin vaccine was developed by the Vaccine Research Center (VRC), National Institute of Allergy and Infectious Diseases (NIAID), which is composed of *Helicobacter pylori* non-heme ferritin assembled with the influenza virus H1 HA insert to form a NanoP displaying eight HA-stabilized stem trimers from A/New Caledonia/20/1999 (H1N1) influenza virus. The phase 1 clinical trial of this universal influenza vaccine candidate is to examine the vaccine’s safety, tolerability, and ability to induce an immune response in healthy volunteers aged 18 to 70 [116]. Wang et al. (2018) predicted that a universal influenza vaccine would be available in eight to 10 years by incorporating layered protein nanoparticles for effective delivery and controlled release technology, like dissolvable microneedle patch-based skin vaccination [110].

## 6. Conclusions

Current seasonal influenza vaccines in use for humans and animals are lacking efficacy and can only provide partial protection (10–60%) against influenza viruses. The worldwide rapid spread of the 2009 influenza pandemic highlighted the urgent need for a cross-protective universal influenza vaccine against different clades of viruses. Advanced vaccine platform technologies have enabled us to achieve significant progress in the design and development of new universal influenza vaccines. Due to the serious constraints of current egg-based influenza vaccine technology, a new generation of egg-free influenza-based vaccines have been actively investigated. Among the newly-developed vaccine technologies, genetic (RNA and DNA), recombinant multi-epitope-based peptide, or VLP vaccines hold special promises. However, these vaccine platforms may suffer from limitations like in vivo stability, delivery, bioavailability, and immunogenicity. The new generation of NanoP-based vaccines showed great potential to address limitations like antigen availability, targeted delivery, and immunogenicity, as well as antigen recognition by the APC. Moving forward, a universal influenza vaccine should induce not only the HA and NA antibodies, but also T cell responses against highly conserved viral antigens. Several new vaccine platforms which targeted conserved influenza antigens like the stem of HA, NA, NP, and M2 have demonstrated broad cross-protection, heterosubtypic immunity, and long-lasting protection in pre-clinical animal models. The most clinically-advanced phase III vaccine candidates are the Biondvax’s M-001 and Medicago’s MT-2271 (VLP-based vaccine). Some of the vaccines comprising conserved viral antigens like MVA-NP/M1, H1-stabilized stem ferritin vaccine, and HA-stem as LAIV adjuvanted with AS03A are currently undergoing clinical trials in humans. Although several of the novel vaccine technology platforms are promising for the development of a universal influenza vaccine, it is obvious that none of the pre-clinical or early clinical trials can mimic the real situation of a pandemic. Therefore, future studies might need to incorporate either inactivated or IIV vaccines for boosting possible heterologous protection. In addition, for newly emerging influenza viruses from pandemics, conserved T cell epitope predictive algorithms could provide rational approaches for the design and development of a multi-epitope-based peptide or expressed as a recombinant subunit vaccine.

## Figures and Tables

**Table 1:** Categories of annual influenza vaccines approved by the Food and Drug Administration (FDA).

Type of Vaccine	Virus Strain	Trade Name	Production	Age	Immunological Outcomes	Route	Ref.
IIV	Influenza A: H1N1 and H3N2 virus/Influenza B: Victoria and/or Yamagata	Fluzone	Eggs	6–35 m	Ab immune response	IM	[34]
		Fluarix	Eggs	>6 m			
		Flucelvax	MDCK cells	>4 y			
RIV	Contains the HA ectodomain amino acid sequence of cell-cultured vaccine prototype viruses suggested by WHO	Flublok	Recombinant-expression in insect cell line	>18 y	Ab immune response	IM	[35,36]
LAIV	Subtypes of H1N1 and H3N2 (influenza A) and one Influenza B	FluMist	Eggs	2–49 y	Mucosal (nasal) IgA Ab and strong cell-mediated immunity	IN	[37,38,39]

Madin–Darby canine kidney (MDCK); intranasal (IN); intramuscular (IM); antibody (Ab); month (m); year (y); inactivated influenza vaccine (IIV); hemagglutinin (HA); recombinant influenza vaccine (RIV); World Health Organization (WHO); live attenuated influenza vaccine (LAIV)
